# The Optimization of Medium Conditions and Auxins in the Induction of Adventitious Roots of Pokeweed (*Phytolacca americana* L.) and Their Phytochemical Constituents

**DOI:** 10.1155/2023/2983812

**Published:** 2023-08-21

**Authors:** Attachai Trunjaruen, Prathan Luecha, Worasitikulya Taratima

**Affiliations:** ^1^Department of Biology, Faculty of Science, Khon Kaen University, Khon Kaen 40002, Thailand; ^2^Department of Pharmacognosy and Toxicology, Faculty of Pharmaceutical Science, Khon Kaen University, Khon Kaen 40002, Thailand

## Abstract

Pokeweed, *Phytolacca americana* L., is considered a widely spreading invasive plant, while saponin contents accumulated in the roots have pharmaceutical uses, such as rheumatism treatments and anti-inflammation. Adventitious root cultures are an important source of diverse secondary metabolites, which have significant applications in various fields. This study focused on the optimization of parameters for root induction using different medium states and treatments with auxins on a pokeweed leaf. Semisolid and liquid MS (Murashige and Skoog, 1962) media were supplemented with indole-3-butyric acid (IBA) and 1-naphthylacetic acid (NAA) at 0.5, 1, 2, and 4 mg/L. Root growth parameters, *e.g.*, induction percentage, root numbers, length, and weight, were measured to determine the adventitious root induction efficiency. Total phenolic content, total flavonoid content, total saponin content, and antioxidant activity were recorded. Results showed that adventitious roots induced in semisolid MS medium supplemented with 0.5 mg/L NAA exhibited a high density of lateral roots. Appropriate medium state and auxin for adventitious root induction in pokeweed were determined as semisolid medium supplemented with 2 mg/L NAA. Considering phytochemicals, adventitious roots induced in liquid medium containing 0.5–1 mg/L NAA had the highest yield extract percentage. Additionally, adventitious roots cultivated in a liquid medium enriched with 1 mg/L NAA exhibited the highest phenolic and saponin contents. A principal component analysis (PCA) biplot and hierarchical cluster analysis (HCA) heatmap demonstrated different response patterns between semisolid and liquid media applied with NAA. The results of the semisolid media were grouped together due to high expression levels of the root induction parameters, while elevated phytochemical values were observed in the liquid media treatments. The results suggested two different media that provide the highest adventitious root induction efficiency and the greatest phytochemical contents: semisolid medium with 2 mg/L NAA and liquid medium with 1 mg/L NAA, respectively. These culture media can be applied to optimize adventitious root culture of pokeweed and *in vitro* phytochemical production.

## 1. Introduction

Pokeweed (*Phytolacca americana* L.) belongs to the Phytolaccaceae family. While native to North America, it has been domesticated in several countries across Europe, South America, Australia, and Asia as an ornamental plant. However, it has also become a widespread invasive species [1]. Pokeweed is also used for cloth coloration, with recent applications as a histological dye because betacyanins accumulated in pokeweed berries provide red and violet dyes [2]. Few reports have addressed pokeweed toxicity. Turkey poult and molluscicide toxic properties were examined by Barnett [3], while young cooked leaves were reported to be consumed as a salad in rural regions of North America [4]. All parts of the pokeweed plant have been utilized as herbal remedies. Pokeweed roots and berry extracts have been used to treat rheumatism [5, 6]. Additionally, pokeweed roots have been employed in the treatment of breast cancer [1]. In China, they have traditionally been used to alleviate edema and treat skin infections [7]. Moreover, pokeweed has shown promise as a noteworthy medicinal plant in the field of cancer therapy.

Although generally considered an invasive species, pokeweed has multiple uses, with various bioactive compounds discovered in different pokeweed parts. Neolignans, such as americanin A and isoamericanol A, isolated from pokeweed seeds showed antibreast and colon cancer activities [8–10]. Pokeweed storage roots contain triterpenoid saponins that possess useful bioactivities, such as anticancer and antifungal activities. Esculentoside A, a triterpenoid saponin isolated from pokeweed roots, has been proposed as a candidate for human breast cancer treatment because of its inhibition effects on breast cancer growth [11, 12]. Esculentoside H has the inhibitory bioactivity against human cancer cell migration [11, 13]. Antifungal bioactivity has also been reported by phytolaccoside B isolated from pokeweed roots [11, 14]. This research showed that pokeweed possesses various compounds in different plant parts, especially in the roots, accompanied by notable bioactivities. However, in field cultivation, it would take almost a year to harvest the mature pokeweed roots [1]. Although pokeweed was not addressed in the conservation red lists of IUCN [15], *in vitro* root culture offers a promising approach to produce high-quality and pathogen-free pokeweed roots, thereby reducing the required cultivation duration. In addition, *in vitro* root culture can be established to produce bioactive compounds in a controlled and sustainable manner without contributing to the spread of invasive species.

Plant tissue culture is generally used for plant propagation under aseptic conditions. This technique is now widely applied in different fields because it can be used under time and space constraints [16]. Plant tissue culture is employed for large-scale production of new whole plants or specific plant parts. These propagated materials, with or without elicitors, can be utilized to extract bioactive compounds. Fruits of *in vitro* grown plants of *Piper longum* and cell suspensions of *Cnidoscolus chayamansa* showed greater contents of piperine and lupeol acetate than *in vivo* plants, respectively [17, 18]. There is a report that investigated the *in vitro* production of betacyanin from pokeweed cell suspension [19]. It was discovered that an elevated sucrose concentration and the application of 2,4-dichlorophenoxyacetic acid (2,4-D) could induce higher betacyanin accumulation in the cell suspension [20, 21]. Additionally, apart from betacyanin, phytolaccoside B, a triterpenoid saponin, was isolated from pokeweed callus cocultured with *Botrytis fabae* [22]. However, *in vitro* propagation of other pokeweed parts, such as roots, and their applications as materials for bioactive compound production are still limited.

Auxin has been used as the key regulator of *in vitro* root induction [23], which several studies have provided evidence supporting its role in this process. In root induction of *Polygonum multiflorum*, indole-3-butyric acid (IBA) was more effective than 1-naphthylacetic acid (NAA) in root biomass, phenolic, and flavonoid production [24], while NAA was suitable for root induction of *Gypsophila paniculata* and the production of saponins when compared to IBA [25]. These studies demonstrated that auxin can be used to establish *in vitro* root culture for phytochemical production, but the appropriate type and concentration of auxin for pokeweeds need to be studied. Additionally, root induction in liquid medium can promote root growth and the production of phytochemicals due to better nutrient circulation than solid medium [26, 27]. Therefore, this study established appropriate medium states and plant growth regulators (PGRs) for *in vitro* pokeweed adventitious root induction from leaf explants, considering root growth and phytochemical parameters. Correlations among growth and phytochemical parameters were also investigated to reveal the response patterns of adventitious roots under different culture media.

## 2. Materials and Methods

### 2.1. Plant Materials

Dry specimens of pokeweed (*Phytolacca americana* L.) were prepared and identified by Asst. Prof. Dr. Sakuntala Ninkaew and then deposited at the Khon Kaen University (KKU) herbarium (KKU No. 26576). Pokeweed berries were harvested from mature plants cultivated in Sakon Nakhon Province, Thailand. The seeds were separated from the pokeberries, and dormancy was broken by soaking in concentrated sulfuric acid for 15 min and rinsing with running tap water. Seed sterilization was performed by shaking with 12% (v/v) Clorox (0.72% v/v sodium hypochlorite) for 45 min and rinsing with sterilized distilled water for five minutes at least three times. The sterilized seeds were germinated on free PGRs and Murashige and Skoog (MS) medium added with 30 g/L sucrose [28]. The pH was adj usted to 5.8 before solidification with 8 g/L agar. The medium was sterilized by autoclaving at 121°C for 20 min. The cultures were maintained at 25 ± 2°C for six weeks under a 16/8 h (light/dark) cycle at 40 *μ*mol·m^−2^·s^−1^ light intensity, and the mature plants were used to provide the initial plant materials.

### 2.2. Adventitious Root Induction

The first two leaves next to the apical shoot tip from 6-week-old plants were harvested and cut into small pieces, 1 × 1 cm^2^ in size, as the initial materials for culture on different medium states. The liquid MS medium was prepared without agar, while the semisolid medium was supplemented with 4 g/L agar. Both medium contained 1-naphthylacetic acid (NAA) or indole-3-butyric acid (IBA) at 0.5, 1, 2, and 4 mg/L. Liquid and semisolid media without PGRs were used as controls. The cultures were maintained at 25 ± 2°C for six weeks under a 16/8 h (light/dark) cycle at 40 *μ*mol·m^−2^·s^−1^ light intensity. The liquid cultures were agitated at 120 rpm.

After four weeks of culture, survival and response percentages were recorded. Explants with adventitious roots were counted to calculate the adventitious root induction percentage. The number of primary and secondary adventitious roots and their length were recorded simultaneously. All adventitious and lateral roots generated from an explant were collected to determine fresh and dry weight. For dry weight, fresh samples were dried at 50°C for three days before data recording. These variables were considered root growth parameters.

### 2.3. Sample Extraction

The dried samples of adventitious roots were ground, and 20 mg of the powder was extracted with 1 ml methanol (MeOH; AR grade) and sonicated at 30°C for 30 min before the extracts were collected and replaced with new MeOH. The sonication was repeated three times. MeOH was evaporated using a vacuum concentrator at 50°C and 100 mTorr for 6 h, and the extracts were lyophilized for 48 h. Extract yield percentages were calculated using the following equation:

(1) $$\begin{array}{c} yield\;percentage=\frac{extract\;weight}{sample\;dry\;weight}\times 100\text{.} \end{array}$$

The obtained extracts were evaluated for phytochemical constituents including total phenolic content (TPC), total flavonoid content (TFC), total saponin content, and antioxidant activity by 2,2-diphenyl-1-picrylhydrazyl (DPPH) assay using the following protocols.

### 2.4. Total Phenolic Content (TPC)

The Folin–Ciocalteu assay was applied to determine the total phenolic content following Mwamatope et al. [29] with some modifications. In brief, the extracts were dissolved in MeOH, and 20 *μ*l was loaded into microplates. Then, 100 ml of 0.2 M Folin–Ciocalteu reagent and 80 *μ*l of 7% (w/v) Na_2_CO_3_ were added. The mixtures were incubated at room temperature under darkness for 30 min. The absorbance was evaluated at 760 nm by a microplate reader (Ensight® Multimode Plate Reader, PerkinElmer, USA). TPC was calculated as *μ*g gallic acid equivalent (GAE)/mg dry weight.

### 2.5. Total Flavonoid Content (TFC)

TFC was determined by the aluminum chloride assay following Pekal and Pyrzynska [30] with some modifications. The extracts were dissolved in MeOH, and 100 *μ*l was filled into microplates and added to 20 *μ*l of 5% (w/v) NaNO_3_ and 35 *μ*l of 10% (w/v) AlCl_3_, before incubation at room temperature under darkness for 30 min. The absorbance was evaluated at 430 nm by the microplate reader. TFC was calculated as *μ*g quercetin equivalent (QE)/mg dry weight.

### 2.6. Total Saponin Content (TSC)

The vanillin-sulfuric assay was carried out to determine the total saponin content. The extracts were dissolved in MeOH, and 250 *μ*l was loaded into test tubes, which were incubated at 75°C for 20 min to remove MeOH. Then, 8% (w/v) vanillin solution at 250 *μ*l and 2.5 ml of 72% (v/v) H_2_SO_4_ were added into the test tubes before incubating in a water bath at 60°C for 20 min. The absorbance was evaluated at 560 nm by a spectrophotometer (GENESYS™ 140/150 Vis/UV-Vis, Thermo Fisher, USA). TSC was calculated as *μ*g oleanolic acid equivalent (OAE)/mg dry weight [31].

### 2.7. Antioxidant Activity

The 2,2-diphenyl-1-picrylhydrazyl (DPPH) assay was used to investigate the antioxidant activity of pokeweed adventitious root extracts. The extracts dissolved with MeOH at 100 *μ*l were mixed with 100 *μ*l of 0.2 mM DPPH reagent and incubated at room temperature under darkness for 30 min. The absorbance was evaluated at 517 nm by the microplate reader. The percentage of free radical scavenging was calculated using the following equation:

(2) $$\begin{array}{c} percentage\;of\;free\;radical\;scavenging=\frac{\left(A_{control}\;–\;A_{sample}\right)}{A_{control}}\times 100\text{,} \end{array}$$

where *A*_control_ = absorbance of the reaction with MeOH and *A*_sample_ = absorbance of the reaction with sample solution.

Linear regression and an equation showing the relation between sample concentration and free radical scavenging percentages were established. The efficiency of antioxidant activity was calculated from the linear equation and presented as IC_50_ [29].

### 2.8. Statistical Analysis

Root growth and phytochemical parameters were recorded with six and three replicates, respectively. To identify the most suitable conditions for adventitious pokeweed root induction, data were analyzed using analysis of variance (ANOVA). Means were compared using least significant difference (LSD; *p*  <  0.05) with Statistix 10 software. Principal component analysis (PCA) was used to determine the relationships between growth and phytochemical parameters. Response patterns due to different culture conditions were further investigated using hierarchical cluster analysis (HCA). PCA and HCA were performed with Origin Pro 2022 software. Data are presented as mean ± standard error (SE).

## 3. Results and Discussion

### 3.1. Effects of Culture Media on the Growth Efficiency of Pokeweed Adventitious Roots

**Figure 1:**
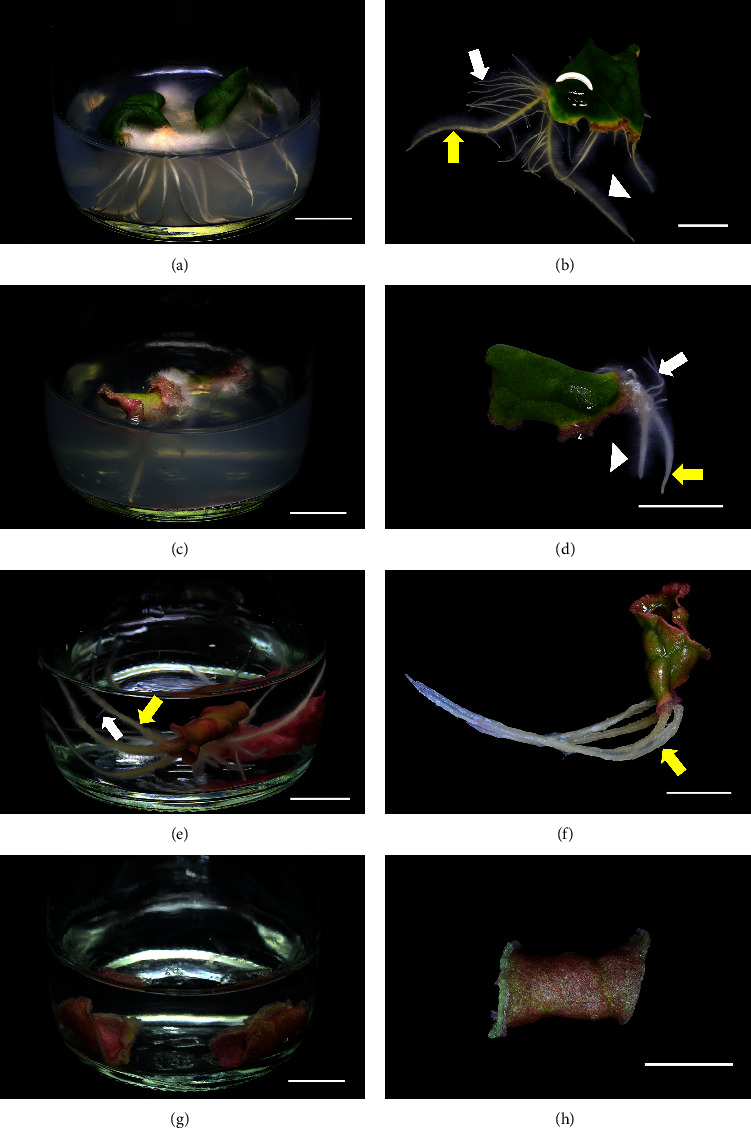
Adventitious root induction from pokeweed leaf explants cultured on semisolid and liquid media containing NAA and IBA: (a) semisolid culture with 2 mg/L NAA; (b) leaf explants with adventitious roots from semisolid culture with 1 mg/L NAA; (c) semisolid culture with 1 mg/L IBA; (d) leaf explant with adventitious roots from semisolid culture with 1 mg/L IBA; (e) liquid culture with 0.5 mg/L NAA; (f) leaf explant with adventitious roots from liquid culture with 1 mg/L NAA; (g) liquid culture with 1 mg/L IBA; (h) leaf explant with adventitious roots from liquid culture with 1 mg/L IBA (yellow arrow = adventitious root; white arrow = lateral root; arrow head = root hair; all scales = 1 cm).

The explants were cultured on semisolid and liquid media supplemented with various concentrations of NAA and IBA for four weeks. The leaf explants achieved 100% survival and responded differently to culture media. No adventitious roots emerged from leaf explants in the semisolid and liquid control treatments, while enlargement of the explants was observed in all treatments. In addition to the enlargement of the cultured leaf explants, the accumulation of purple pigment was also observed, particularly in liquid culture. The accumulated pigments could be betacyanins, the major purple pigments in pokeweed, which their contents could be improved using exogenous auxin [21]. Pokeweed adventitious roots directly emerging from leaf explants were observed in all semisolid and NAA treatments of liquid media, with differences in root morphology. Adventitious roots from semisolid media with NAA and IBA had dense root hairs with a large number of lateral roots developing from the adventitious roots (Figures 1(a)–1(d)), while roots from NAA and IBA in semisolid media had different lengths. In contrast to solid media, liquid media supplemented with NAA induced sparse root hairs with small numbers of lateral roots (Figures 1(e) and 1(f)), while IBA did not produce any adventitious roots (Figures 1(g) and 1(h)).

**Figure 2:**
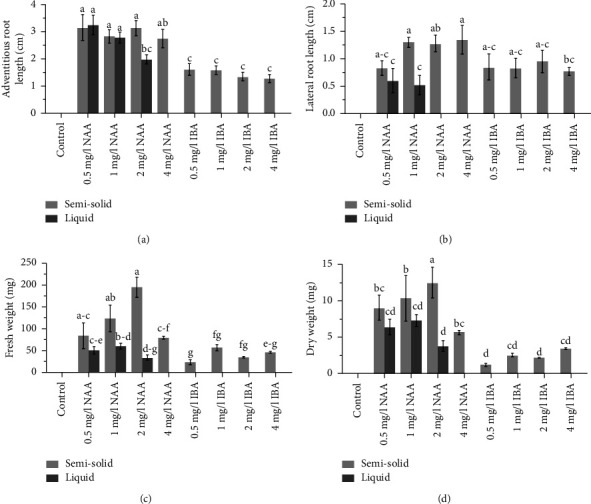
Length of adventitious and lateral roots (a, b) and fresh and dry weight of pokeweed roots (c, d) obtained from semisolid and liquid cultures with NAA and IBA. Data are represented by means and bars represent ± SE, with different letters indicating significant differences by two-way ANOVA and LSD (*p*  <  0.05).

Among all culture treatments, semisolid MS media containing NAA and IBA provided adventitious roots, with root induction percentages ranging from 58.33% to 91.67%. On the other hand, liquid media supplemented with 0.5–2 mg/L NAA produced adventitious roots with induction percentages ranging from 50.00% to 100.00%. The highest percentage was recorded with 1 mg/L NAA in liquid medium (100.00%) and was not significantly different from semisolid media with 1–4 mg/L NAA and liquid medium with 0.5 mg/L NAA (Table 1; *p*  >  0.05). IBA-supplemented semisolid and liquid media had lower effectiveness of adventitious root induction than NAA. Liquid media containing 0.5–2 mg/L NAA provided a significantly larger number of adventitious roots (5.83–6.17 roots per explant) than the other treatments (Table 1; *p*  <  0.05). Only a few lateral roots were recorded in liquid media, while numerous lateral roots were observed in semisolid media. The treatment of 0.5 mg/L NAA in semisolid medium gave the highest number of lateral roots, which decreased when NAA concentration increased (Table 1). The longest adventitious roots, 2.75–3.23 cm, were obtained from semisolid media with NAA and liquid media with 0.5–1 mg/L NAA, while the longest lateral roots were harvested from 1 to 4 mg/L NAA treatment in semisolid media (Figures 2(a) and 2(b); *p*  <  0.05). The fresh weight of the obtained roots demonstrated the same trend as the dry weight. The highest fresh and dry weights were obtained from 2 mg/L NAA in semisolid medium (Figure 2(c); *p*  <  0.05), while liquid media containing NAA gave lower weights than similar NAA concentrations in semisolid media (Figure 2(d)). Therefore, treatment of semisolid medium fortified with 2 mg/L NAA was optimal for pokeweed adventitious root induction, providing high adventitious root induction, root length, and root biomass.

Auxin is considered a key factor and the most common PGR for *in vitro* root induction [23]. Roots grown under *in vitro* conditions are technically considered adventitious roots because they do not develop from primary roots [32]. Adventitious root growth occurs in three stages: induction, initiation, and expression, and several genes involved in these phases are regulated by auxin [33]. However, not all auxins have the ability to induce adventitious root development. Trunjaruen et al. [34] compared the effects of IBA and 2,4-dichlorophenoxyacetic acid (2,4-D) on pokeweed micropropagation. Their results showed that pokeweed roots were not observed in 2,4-D treatments. Therefore, only the effects of NAA and IBA were investigated to identify the optimal conditions for adventitious root induction from pokeweed leaf explants.

Solidifying agents provide support for explants and are a necessary factor for root induction. However, too much solid medium inhibits root elongation because roots require space and a loose, aerated environment to grow and expand. Therefore, excessive solid medium can restrict root movement and limit elongation [35]. In this study, 4 g/L agar as a semisolid medium was applied and compared with liquid medium for pokeweed adventitious root induction. Findings demonstrated that semisolid medium was appropriate for adventitious root induction depending on most growth parameters because all semisolid media provided adventitious roots from pokeweed leaf explants. When comparing liquid and solid media, *Stemona curtisii* and two cultivars of *Solanum tuberosum* showed effective root induction in solid medium [36, 37], while adventitious root induction of *Ananas comosus* was performed in liquid medium [38]. The appropriate medium state for adventitious rooting depends on the plant genotype. Most previous research on adventitious root induction was conducted with gelled medium, such as the adventitious root induction of *Argania spinosa* and *Morinda citrifolia* from shoots and leaf explant, respectively [39, 40], because the solidified medium provides a supporting material and acts as a pH stabilizer [41]. Our results also showed denser lateral roots and root hairs on adventitious roots in the semisolid medium. Semisolid media play major roles in plant anchorage and facilitate water uptake [35]. Interestingly, among the growth parameters, only the number of pokeweed adventitious roots obtained from liquid medium was higher, while all other parameters were optimal in semisolid medium. This phenomenon resulted from higher diffusion rates of liquid nutrients into the explants [25]. However, root morphology in semisolid medium (dense root hairs and lateral roots) was more appropriate for plant acclimatization.

When evaluating the effects of auxins in the media, the results showed that NAA produced higher adventitious root induction effectiveness than IBA. The use of NAA resulted in larger number of adventitious and lateral roots with increased lengths as well as higher root fresh and dry weights. The effects of NAA in adventitious root induction have been proven in several plant species. The most effective root culture of *Phyllanthus urinaria* was established using medium with NAA [42]. The application of a medium enriched with 5 mg/L of NAA led to higher fresh and dry weights of *Morinda citrifolia* adventitious roots compared to those obtained from treatments of IBA [40]. Likewise, 5 mg/L NAA promoted adventitious root induction from stem cuttings of *Bienertia sinuspersici* [43]. IBA efficiency was lower than NAA, but our results indicated that IBA in semisolid medium initiated adventitious roots, with similar results observed in *Musa acuminata* [44], *Solanum procumbens* [45], and *Dillenia suffruticosa* [46]. These studies proved that various plant species or explants require specific PGRs and culture media to initiate adventitious roots. Several studies have combined NAA with other PGRs, while exogenous auxins, especially NAA, are key regulators necessary for the induction of adventitious roots. Application of NAA alters the balance of indole-3-acetic acid (IAA), which is an endogenous and natural auxin associated with adventitious root initiation. In the adventitious root culture of *Hemarthria compressa*, higher concentrations of NAA led to an increase in IAA levels. This increase in IAA was reflected by a decrease in the activity of indole-3-acetic acid oxidase (IAAO). Moreover, positive correlations between NAA and IAA concentrations were observed in the adventitious root induction of *Populus* hybrids [47, 48]. Elevated IAA levels promote the division of adventitious root founder cells and the elongation of interfascicular cambium in the induction and initiation phases of adventitious root development [49]. During the induction phase, NAA induces root founder cells by the degradation of *AUXIN/INDOLE-3-ACETIC ACID* (*AUX/IAA*) family proteins. The degradation allows the *AUXIN RESPONSE FACTOR* (*ARF*) gene family to upregulate *GRETCHEN3* (*GH3*) genes and promote adventitious root formation [32, 33]. During adventitious root initiation, the application of NAA led to the activation of *WUSCHEL-RELATED HOMEOBOX 11* and *12* (*WOX11/12*) genes. Subsequently, downstream genes like *WOX5/7* and *LATERAL ORGAN BOUNDARIES DOMAIN 16* (*LBD16*) were induced, leading to the formation of root primordium cells [33, 50]. However, some studies found that NAA could not induce an increase in IAA content because NAA directly promoted cell division and elongation *via* regulation of gene-associated root apical meristem initiation [51]. Consequently, exogenous NAA and IBA are effective for the establishment of pokeweed adventitious root culture from leaf explants, while 2 mg/L NAA in semisolid medium is better for pokeweed adventitious root induction.

### 3.2. Phytochemical Constituents and Antioxidant Activity of the Adventitious Roots

Adventitious roots were extracted with MeOH to evaluate and compare their phytochemical constituents with wild pokeroots. Results showed a remarkably high yield percentage of crude extracts of liquid media fortified with NAA, ranging from 35.84% to 45.63% and significantly higher than all other treatments of semisolid media and 8-month-old wild roots collected form Sakon Nakhon, Thailand (Table 2; *p*  <  0.05). Interestingly, semisolid media with IBA also provided a higher yield percentage of crude extracts than NAA-containing semisolid media.

TPC, TFC, and TSC determinations revealed outstanding contents of pokeweed adventitious roots. Liquid media with 0.5–1.0 mg/L NAA provided higher TPC, TFC, and TSC than the wild root and semisolid media at the same NAA concentration. Interestingly, semisolid media containing IBA showed elevated TPC, TFC, and TSC over semisolid media with NAA. The highest TPC and TSC were obtained from the treatment of 1 mg/L NAA in liquid medium, while 0.5 mg/L IBA in semisolid medium produced the highest TFC (Table 2; *p*  <  0.05).

The antioxidant activities of pokeweed adventitious root extracts were evaluated using DPPH, with results presented as IC_50_. Results showed that liquid media fortified with NAA gave significantly lower IC_50_ of the DPPH assay (*p*  <  0.05), ranging from 753.46 to 1045.13 *μ*g/ml. The lowest IC_50_ (753.46 *μ*g/ml) was achieved from the treatment of liquid medium with NAA at 2 mg/L, while the other treatments gave over 1280 *μ*g/ml, with results out of the linearity range (Table 2). Consequently, liquid medium supplemented with 1 mg/L NAA was the most appropriate for pokeweed adventitious root induction and bioactive compound accumulation, recording the highest TPC and TSC and the most effective antioxidant activity.

Several previous studies reported positive effects of PGRs on *in vitro* production of phytochemicals in solid, semisolid, and liquid cultures, such as *Heliotropium indicum* leaves [52], *Eryngium alpinum* multiple shoots [53], and *Polygonum multiflorum* adventitious roots [23]. Our results revealed that liquid media containing 0.5–2 mg/L NAA and semisolid media with individual NAA or IBA provided pokeweed MeOH extracts and elevated the phytochemical contents of pokeweed adventitious roots. However, the interaction effects between different auxins and auxin with other PGRs on pokeweed adventitious root induction and the phytochemical contents from the induced roots need to be further studied.

Our results demonstrated that semisolid media containing IBA gave higher yield percentage, TPC, TFC, and free radical scavenging activity than treatment of NAA in semisolid media. The superior efficiency of IBA for *in vitro* production of phenolic and flavonoid compounds over NAA was also observed in adventitious root cultures of *Polygonum multiflorum* [23]. More effective results of IBA on phenolic compound contents were also found in *Gnetum buchholzianum* roots [54]. However, plant species are selective for optimal PGR and IBA choices. Media containing NAA with or without other PGRs provided elevated accumulated TPC and TFC in *in vitro* grown leaves of *Heliotropium indicum* [52], while *in vitro* roots of *Salvia miltiorrhiza* showed higher TPC accumulation than seed-derived plants [55]. The developmental stages of explants also showed variation in plant responses to PGRs for TPC accumulation. Emile et al. [54] revealed that exogenous IAA resulted in the highest TPC at the initiation stage of *Gnetum buchholzianum* root development, while IBA was more effective than other auxins at the expression stage. Plant species and developmental stages respond to PGRs differently, confirming that exogenous auxins improve the accumulation of phenolic compounds. Several studies have shown that phenolic accumulation in induced roots is associated with interactions among some oxidase enzymes. Polyphenol oxidase (PPO) is involved with the oxidation of monophenol to diphenol and quinones [56], leading to elevated phenolic acid accumulation. *In vitro* roots of *Cotinus coggygria* treated with IBA demonstrated a positive correlation between PPO activity and polyphenolic acid contents [57], while combined treatment of IBA and NAA considerably improved some phenolic acid contents, such as chlorogenic and coumaric acid, in *Magnolia* hybrid roots [58]. These phenolic acids are intermediates in lignin biosynthesis regulated by peroxidase (POX), which is necessary for cell wall genesis in the late developmental stage of adventitious rooting [46, 57]. Phenolic compounds form complexes with IAA to prevent IAA decarboxylation [54, 59]. These studies confirmed the interaction between PPO and POX in rooting and phenolic compound accumulation, responding patterns, and dynamic changes of these enzymes to exogenous auxins depending on plant species [56]. Consequently, exogenous auxins can promote the accumulation of phenolic compounds in adventitious roots through the regulation of PPO and POX activities.

In semisolid treatments, the phytochemical contents of media containing IBA tended to be higher than NAA. However, it was observed that liquid medium with 0.5–2 mg/L NAA provided significantly higher phytochemical contents, especially phenolic and saponin contents. In contrast, liquid media containing IBA did not induce the formation of adventitious roots. The highest TPC in liquid medium with NAA also gave the lowest IC_50_ of the DPPH assay, while other media did not show antioxidant potential. Some phenolic acids and flavonoids accumulated in *Spiraea betulifolia* ssp. *aemiliana* microshoots, like chlorogenic acid and quercetin, were higher than those in microshoots under solid treatment [60]. *In vitro* production of primulic acid I and II from adventitious roots of *Primula veris* ssp. *veris* was established in liquid culture systems with agitation [26]. The main cause of the positive effects of liquid culture on phytochemical production is the close contact between the explants and the medium, thereby providing more available nutrients than solid culture [25, 60]. However, liquid medium supplemented with 4 mg/L NAA or 0.5–4 mg/L IBA had negative effects on pokeweed adventitious rooting and phytochemical contents, possibly because of explant contact with excessive levels of exogenous auxins. Liquid medium with 0.5–2 mg/L NAA also gave the highest saponin contents. The effects of exogenous auxins on elevated saponin contents have been demonstrated in several plant species. Aculeatiside A and B accumulations in the callus of *Solanum aculeatissimum* were enhanced in solid medium with 0.1 mg/L NAA [61]. Furthermore, the application of exogenous auxins induced higher total saponins and saponin glycoside contents in the roots of *Panax ginseng* [62], *Gypsophila paniculata* roots [24], and *Bacopa floribunda* micropropagated plantlets [63]. Auxins can also show positive effects on the phenolic production in root cultures. In the adventitious root induction of *Camellia sinensis* from node cutting, IBA provided the largest number of roots, longest roots, and also elevated total phenolic contents from the induced roots [64]. This recent study confirmed that exogenous auxins had positive effects on *in vitro* phytochemical production, and liquid MS medium with 1 mg/L NAA was the most effective for phenolic, flavonoid, and saponin accumulation in pokeweed adventitious roots.

### 3.3. Correlations between Growth and Phytochemical Parameters Assessed by Principal Component Analysis

**Figure 3:**
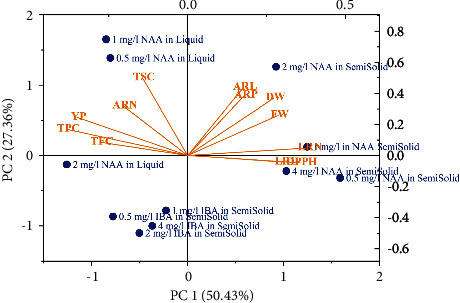
PCA biplot showing the relation among parameters and their influence on the treatments (ARP = adventitious root induction percentage; ARN = adventitious root number; ARL adventitious root length; LRN = lateral root number; LRL = lateral root length; FW = fresh weight; DW = dry weight; YP = yield percentage; TPC = total phenolic content; TFC = total flavonoid content; TSC = total saponin content; DPPH = antioxidant activity by DPPH assay).

Data from treatments providing adventitious roots were analyzed by PCA to reveal the relations among determined parameters and group the treatments according to those parameters. A biplot constructed from PC1 and PC2 explained the data linkage at 77.79%. PC1 accounted for 50.43% of the relation, with separation of the treatments into two clusters depending on the two parameter groups. Positive correlations among the number of adventitious roots, yield percentage, TPC, TFC, and TSC caused the cluster of liquid media containing NAA and semisolid media containing IBA to be plotted in the two left-side quadrants. The other growth parameters and antioxidant activity by DPPH assay, separating the treatments of semisolid media with NAA, were plotted in the two right-side quadrants (Figure 3).

PC2 explained the variance of the relation at 27.36%, enabling discrimination between liquid media with NAA and semisolid media with IBA. Liquid media with 0.5–1.0 mg/L NAA were positioned in the upper left-side quadrants due to their high contents of phytochemicals, yield percentage, and number of adventitious roots. Semisolid media containing NAA were also divided into two groups. Based on the percentage of adventitious root induction, adventitious root length, and fresh and dry weights, the semisolid medium supplemented with 2 mg/L NAA was positioned in the upper right-side quadrant. On the other hand, the other semisolid media fortified with NAA were plotted in the lower right-side quadrant due to the lateral root number, length, and antioxidant activity capacity (Figure 3).

The opposite position between the parameters of root growth and phytochemicals clearly exhibited the negative correlation, which was also observed in *Lactuca sativa* [65] and *Hordeum vulgare* [66]. The negative correlations may be indirectly resulted from the solidification states of the culture. Gelling agents in semisolid media provide supporting material and stimulate lateral roots and root hairs to absorb water [35, 36], leading to high values in growth parameters. However, the treatments with semisolid media provided lower response in phytochemical parameters because liquid culture served more nutrients and PGRs than semisolid media. Additionally, the negative correlation of TPC and TFC with DPPH antioxidant activity could be explained by the radical scavenging efficiency of the compounds. The extract with high phenolic and flavonoid contents always provides high antioxidant activity, resulting in the lower IC_50_ of DPPH assay. These evidences supported the inverse relation between phytochemical and root growth parameters in both direct and indirect ways.

The PCA results obviously revealed that the culture on liquid medium and the addition of NAA positively affected the parameters of adventitious root number, phytochemicals, and yield percentage. The relations were exhibited by the same plotting position of these parameters and the treatments of liquid media with NAA (Figure 3). In addition to the adventitious induction, the effects of NAA on the elevated TPC have been proved in *Salvia miltiorrhiza* [55]. The application of exogenous auxins has demonstrated the improved content of total saponin in several plant species [62, 63]. The efficiency of NAA in influencing these phytochemical contents may be attributed to the increased enzyme activities associated with the biosynthesis pathways of these compounds [56]. Moreover, the culture on liquid medium also caused the synergistic effects with fortified NAA on the phytochemical production and root induction because liquid medium could provide close contact and high nutrient absorption of the explants [25, 60]. Therefore, the synergistic effects resulted in the cluster of the liquid media with NAA treatments depending on the phytochemical parameters, yield percentage, and adventitious root number. Although the treatments of liquid media with NAA were influenced by phytochemical parameters and adventitious root number, the treatments of liquid medium with 2 mg/l NAA were discriminated from the others. The discrimination resulted from the weak relation between the treatment and TSC as displayed by their plotting in different quadrants (Figure 3).

The other group was clustered by six root growth parameters and DPPH antioxidant activity. This group included the treatments of semisolid media supplemented with NAA. The positive effects of NAA on the adventitious root growth were synergistically influenced by the semisolid state of the media. These results from the fact that the semisolid medium offers supportive materials for effective root anchoring, simultaneously promoting the growth of lateral roots and root hairs that are necessary to facilitate water uptake from the medium [35, 41]. However, it is important to note that such developmental features might not be essential for root cultivation in the liquid medium. Consequently, the semisolid medium state and exogenous NAA synergistically affect adventitious and lateral root growth. Moreover, the positive relation between six root growth parameters and the treatments of semisolid media with NAA allowed them to cluster together. Noticeably, the treatment of semisolid medium containing 2 mg/l NAA was slightly separated from the others because the treatment showed stronger positive correlation with the adventitious root growth parameters (ARN and ARP) and biomass (FW and DW).

### 3.4. Heatmap and HCA Showing the Growth and Phytochemical Response Patterns of the Culture Treatments

**Figure 4:**
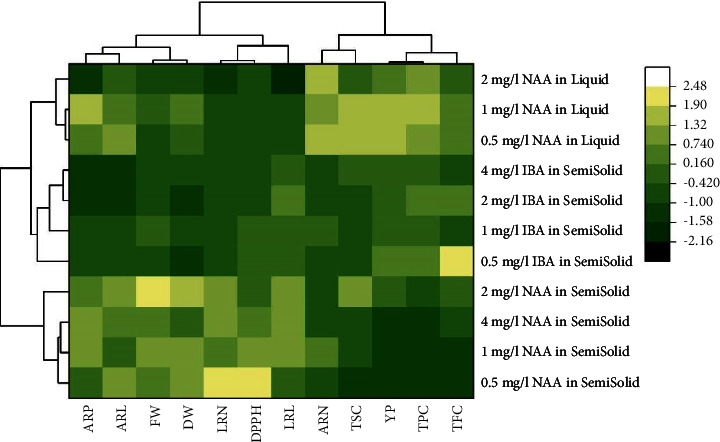
The HCA heatmap explaining the response patterns of growth and phytochemical constituents due to different culture treatments (ARP = adventitious root induction percentage; ARN = adventitious root number; ARL adventitious root length; LRN = lateral root number; LRL = lateral root length; FW = fresh weight; DW = dry weight; YP = yield percentage; TPC = total phenolic content; TFC = total flavonoid content; TSC = total saponin content; DPPH = antioxidant activity by DPPH assay).

The heatmap created from HCA revealed the growth and phytochemical response patterns of all culture treatments. Results confirmed the clustering data from the PCA biplot. All treatments were separated into three groups based on the response patterns of the determined parameters. The first group consisted of semisolid media with NAA, which demonstrated high expression levels of most growth parameters and antioxidant activity (yellowish), with decreased expression levels observed in adventitious root number and phytochemical constituents (greenish). By contrast, liquid media with NAA were grouped together depending on the high content of yield percentage, TPC, TFC, TSC, and adventitious root number, while other parameters were lower than the first group mentioned above. Semisolid media containing IBA revealed the average expression levels of all parameters that were clustered separately from the first two groups (Figure 4).

Root growth and phytochemical parameters were further analyzed to reveal their relation between treatments. The results from HCA were observed to be consistent with the data distribution in a PCA biplot. The PCA biplot showed that most growth parameters were inversely related to phytochemical parameters. The HCA heatmap revealed the clustering of the collected parameters into two clusters: (1) yield percentage, TPC, TFC, TSC, and adventitious root number and (2) six root growth parameters and DPPH. Similar indirect relations between biomass and phytochemical contents were also observed in *Lactuca sativa* [65] and *Prunus persica* fruits [67]. Gelling agents provide supporting material and stabilize the medium pH [35, 36], thereby promoting high pokeweed adventitious and lateral rooting induction in semisolid media treatments, especially with NAA.

The HCA heatmap showed that all treatments of semisolid media containing NAA were clustered depending on the high responding levels in root growth parameters, but they showed declined response in phytochemical parameters. Interestingly, the adventitious roots induced from semisolid media with 2 mg/L NAA had strongly positive trends with adventitious root induction percentage (ARP) and adventitious root length (ARL), indicating that the root culture medium resulted in the optimal percentage and longest roots.

The effects of different auxins were also apparent in the PCA biplot. Semisolid cultures supplemented with IBA were grouped together in the HCA heatmap. This grouping was a result of the moderately negative effects observed on root growth and phytochemical parameters. The results were consistent with the PCA biplot, showing the opposite positions between this group and all parameters. However, the remarkable differences were observed in the treatment of liquid media supplemented with NAA, where their phytochemical contents and adventitious root numbers were strongly positively correlated with phytochemical parameters. Recent findings confirmed that exogenous auxins improved some phytochemical contents through the regulation of endogenous auxin balance, enzyme activity, and gene expression [46, 56]. Consistent with the PCA biplot, the results from the heatmap showed high responses of phytochemical parameters in liquid cultures with NAA. However, the culture under the conditions was not appropriate for adventitious root induction, as reflected by the low response of growth parameters. Interestingly, a negative correlation between antioxidant activity and some phytochemicals, including phenolics and flavonoids, was also demonstrated by the PCA biplot. Antioxidant properties are usually expressed as IC_50_, with low values indicating the high potential of antioxidants. Phenolic and flavonoid compounds are responsible for the high efficiency of antioxidant activity because they act as electron donors to reactive oxygen species (ROS) and prevent biological molecules from oxidizing the chain reaction [68]. Similar relations between phenolic compounds and antioxidant activities were also demonstrated in *Isodon rugosus* calluses [69], *Daucus carota* roots [70], and *Beta vulgaris* leaves [71].

Our results demonstrated that root growth parameters had negative relationship with phytochemical parameters as illustrated by the opposite direction between them in a PCA biplot. The parameters also caused the response trends in pokeweed roots induced in various treatments, which was further explained by the HCA heatmap. The results of the HCA and heatmap, thus, elucidate the relationship among pokeweed adventitious roots from different media depending on the responding patterns of the collected parameters. The finding indicated that the highest response in root growth parameters was expressed in the adventitious roots from semisolid media with NAA. On the other hand, the roots from liquid media with NAA exhibited the highest response in phytochemical parameters. These observations help to understand how different media compositions affect the growth and phytochemical profiles of pokeweed adventitious roots.

## 4. Conclusion

Pokeweed roots contain several bioactive compounds, especially saponins. However, overexploitation of pokeweed roots threatens the viability of their natural population. This study focused on pokeweed adventitious root induction and *in vitro* phytochemical production using different medium states and exogenous auxins. Results suggested that semisolid MS medium fortified with 2 mg/L NAA was the most suitable medium for adventitious root induction, while liquid MS medium supplemented with 1 mg/L NAA was optimal for *in vitro* production of phenolic compounds and saponins from adventitious roots, providing higher phytochemical contents than *ex vitro* cultivated roots. The PCA results revealed the negative relationship between root growth and phytochemical parameters. The parameters also caused the response trends in pokeweed roots induced in various treatments. The heatmap of HCA further explained the relationship of pokeweed adventitious roots from various treatments depending on the collected parameters and indicated that the highest response in root growth and phytochemical parameters was expressed in the adventitious roots from semisolid media with NAA and liquid media with NAA, respectively. The results of this study determine the solidification state of the medium and identified the most suitable auxins to induce pokeweed adventitious roots with the highest phytochemical contents. However, the interaction between auxin and other PGRs, including other factors, needs to be further studied to provide promising conditions for the *in vitro* phytochemical production from pokeweed roots.

## Figures and Tables

**Table 1:** Pokeweed adventitious root induction percentages and number of adventitious and lateral roots influenced by medium state and supplemented PGRs.

Treatment	Adventitious root induction percentage (%)		Number of adventitious roots (roots per explant)		Number of lateral roots (roots per explant)	
	Semisolid	Liquid	Semisolid	Liquid	Semisolid	Liquid
Control	0.00 ± 0.00^f^	0.00 ± 0.00^f^	0.00 ± 0.00^e^	0.00 ± 0.00^e^	0.00 ± 0.00^d^	0.00 ± 0.00^d^
NAA (mg/L)						
0.5	75.00 ± 17.08^b–d^	83.33 ± 10.54^a–c^	3.00 ± 0.36^b–d^	6.00 ± 0.89^a^	12.67 ± 1.74^a^	2.33 ± 0.71^c^
1	91.67 ± 8.33^ab^	100.00 ± 0.00^a^	4.33 ± 0.80^b^	5.83 ± 0.65^a^	6.50 ± 0.99^b^	1.50 ± 0.55^cd^
2	83.33 ± 10.54^a–c^	50.00 ± 0.00^e^	2.83 ± 0.48^cd^	6.17 ± 0.70^a^	7.33 ± 0.80^b^	0.00 ± 0.00^d^
4	91.67 ± 8.33^ab^	0.00 ± 0.00^f^	2.83 ± 0.65^cd^	0.00 ± 0.00^e^	8.16 ± 1.01^b^	0.00 ± 0.00^d^

IBA (mg/L)						
0.5	66.67 ± 10.54^c–e^	0.00 ± 0.00^f^	2.67 ± 0.56^d^	0.00 ± 0.00^e^	1.67 ± 0.49^cd^	0.00 ± 0.00^d^
1	66.67 ± 10.54^c–e^	0.00 ± 0.00^f^	4.17 ± 0.60^b^	0.00 ± 0.00^e^	2.17 ± 0.60^c^	0.00 ± 0.00^d^
2	58.33 ± 8.33^de^	0.00 ± 0.00^f^	2.67 ± 0.71^d^	0.00 ± 0.00^e^	1.33 ± 0.21^cd^	0.00 ± 0.00^d^
4	58.33 ± 15.37^de^	0.00 ± 0.00^f^	2.83 ± 0.60^cd^	0.00 ± 0.00^e^	1.83 ± 0.15^c^	0.00 ± 0.00^d^

Data are expressed as mean ± SE. Significant differences between means were determined by two-way ANOVA and the least significant difference test (*p*  <  0.05). Different letters (a, b, c,…) represent significant differences in both semisolid and liquid columns (*p*  <  0.05).

**Table 2:** Phytochemical constituents and antioxidant activity by DPPH assay of pokeweed adventitious roots impacted by medium states and supplemented PGRs.

Treatment	Yield percentage (%)			TPC (*μ*g GAE/mg DW)			TFC (*μ*g QE/mg DW)		
	Semisolid	Liquid		Semisolid	Liquid		Semisolid		Liquid
Wild pokeroots	15.59 ± 0.33^de^			1.94 ± 0.02^h^			0.75 ± 0.01^e^		
Control	ND	ND		ND	ND		ND		ND
NAA (mg/L)									
0.5	11.98 ± 0.19^e^	45.06 ± 0.70^a^		0.85 ± 0.02^j^	8.74 ± 0.07^c^		0.62 ± 0.02^e^		2.88 ± 0.28^b^
1	16.04 ± 0.31^d^	45.63 ± 0.96^a^		1.42 ± 0.00^i^	9.96 ± 0.09^a^		0.75 ± 0.05^e^		2.84 ± 0.06^b^
2	30.66 ± 0.50^c^	35.84 ± 1.51^b^		3.87 ± 0.05^g^	9.10 ± 0.09^b^		2.18 ± 0.05^c^		2.19 ± 0.29^c^
4	14.97 ± 0.60^de^	ND		1.97 ± 0.01^h^	ND		1.09 ± 0.01^de^		ND
IBA (mg/L)									
0.5	35.87 ± 2.64^b^	ND		6.30 ± 0.04^d^	ND		4.66 ± 0.08^a^		ND
1	28.52 ± 2.02^c^	ND		4.86 ± 0.04^f^	ND		1.25 ± 0.02^d^		ND
2	30.62 ± 1.80^c^	ND		6.32 ± 0.07^d^	ND		2.30 ± 0.38^c^		ND
4	28.98 ± 0.76^c^	ND		5.88 ± 0.08^e^	ND		1.41 ± 0.11^d^		ND

Treatment	TSC (*μ*g OAE/mg DW)					IC_50_ of DPPH (*μ*g/ml)			
	Semisolid		Liquid			Semisolid		Liquid	
Wild pokeroots	18.00 ± 0.07^g^					>1280^*∗*^			
Control	ND		ND			ND		ND	

NAA (mg/L)									
0.5	14.89 ± 0.04^h^		36.66 ± 0.43^b^			>1280^*∗*^		1013.79 ± 1.78^b^	
1	18.89 ± 0.22^f^		39.35 ± 0.09^a^			>1280^*∗*^		1045.13 ± 49.99^b^	
2	33.84 ± 0.27^c^		24.24 ± 0.08^d^			>1280^*∗*^		753.46 ± 1.33^c^	
4	19.40 ± 0.07^f^		ND			>1280^*∗*^		ND	

IBA (mg/L)									
0.5	17.96 ± 0.27^g^		ND			>1280^*∗*^		ND	
1	18.17 ± 0.08^g^		ND			>1280^*∗*^		ND	
2	18.89 ± 0.32^f^		ND			1208.86 ± 2.77^a^		ND	
4	20.58 ± 0.45^e^		ND			>1280^*∗*^		ND	

Data are expressed as mean ± SE. Significant differences between means were determined by two-way ANOVA and the least significant difference test (*p*  <  0.05). ND denotes not detected. Different letters (a, b, c,…) represent significant differences in both semisolid and liquid columns (*p*  <  0.05). The asterisk (^*∗*^) indicates means that are out of the linearity range.

## Data Availability

The data used to support the findings of this study are available from the corresponding author upon request.

## Supplementary Materials

Supplementary Materials Raw data of pokeweed.
