# Supplementary material for: The Optimization of Medium Conditions and Auxins in the Induction of Adventitious Roots of Pokeweed (Phytolacca americana L.) and Their Phytochemical Constituents
Source: Scientifica (Cairo). 2023 Aug 21;2023:2983812. doi: 10.1155/2023/2983812 (PMC10462441; doi:10.1155/2023/2983812)
Supplement: Supplementary Materials — Raw data of pokeweed. [file 2983812.f1.pdf]

Treatments	Survival	Response	Root inductio	Adv_root_n	Lat_root_nu	Adv_root_len	Lat_root_len	FW	DW
0 mg/l in Sem	100	100	0	0	0	0	0	0	0
0 mg/l in Sem	100	100	0	0	0	0	0	0	0
0 mg/l in Sem	100	100	0	0	0	0	0	0	0
0 mg/l in Sem	100	100	0	0	0	0	0	0	0
0 mg/l in Sem	100	100	0	0	0	0	0	0	0
0 mg/l in Sem	100	100	0	0	0	0	0	0	0
0.5 mg/l NAA	100	100	100	2	14	4.2	1.3	38.8	8.3
0.5 mg/l NAA	100	100	0	2	9	2.2	0.6	196.9	16.1
0.5 mg/l NAA	100	100	100	4	8	5	1.2	55.5	7
0.5 mg/l NAA	100	100	50	4	12	2.7	0.4	45.7	12
0.5 mg/l NAA	100	100	100	3	20	2.5	0.8	17.8	6.2
0.5 mg/l NAA	100	100	100	3	13	2.3	0.7	152.1	4.6
1 mg/l in NAA	100	100	100	5	6	2.7	1.2	75.2	5.8
1 mg/l in NAA	100	100	50	7	6	2.8	1.7	100.7	6.9
1 mg/l in NAA	100	100	100	4	7	2.3	1.3	11.8	23.2
1 mg/l in NAA	100	100	100	4	11	3	1.4	153.4	15.6
1 mg/l in NAA	100	100	100	1	4	3.9	1	200.3	2.4
1 mg/l in NAA	100	100	100	5	5	2.2	1.2	200.3	8.2
2 mg/l NAA in	100	100	100	2	8	3.6	1.8	87.1	15
2 mg/l NAA in	100	100	100	2	10	4.1	1.4	185	16.6
2 mg/l NAA in	100	100	50	5	9	2.6	1.5	252.4	18.2
2 mg/l NAA in	100	100	50	3	6	2.2	1.2	219.1	12.4
2 mg/l NAA in	100	100	100	3	6	3.3	1	235.2	7.1
2 mg/l NAA in	100	100	100	2	5	3	0.8	185	5.5
4 mg/l NAA in	100	100	50	1	10	3.8	2.3	79.5	4.9
4 mg/l NAA in	100	100	100	1	11	1.7	1.9	74.8	6.2
4 mg/l NAA in	100	100	100	4	5	3	1.3	98.4	6.3
4 mg/l NAA in	100	100	100	3	7	3.5	0.6	72.8	4.7
4 mg/l NAA in	100	100	100	5	6	2	0.9	76	6.1
4 mg/l NAA in	100	100	100	3	10	2.5	1.1	80.3	5.64
0.5 mg/l IBA i	100	100	100	1	2	2.4	0.6	24.05	1.325
0.5 mg/l IBA i	100	100	100	2	4	1.2	1.7	24.05	1.325
0.5 mg/l IBA i	100	100	50	3	1	2.2	1.4	35.525	1.775
0.5 mg/l IBA i	100	100	50	3	1	1.3	0.2	35.525	1.775
0.5 mg/l IBA i	100	100	50	2	1	1.4	0.8	9.325	0.6
0.5 mg/l IBA i	100	100	50	5	1	1.2	0.4	9.325	0.6
1 mg/l IBA in	100	100	50	2	2	1.8	1.7	78.7	3.325
1 mg/l IBA in	100	100	100	6	1	2	0.5	78.7	3.325
1 mg/l IBA in	100	100	100	5	4	2.1	0.6	50.2	2.475
1 mg/l IBA in	100	100	50	5	1	1.1	0.9	50.2	2.475
1 mg/l IBA in	100	100	50	4	4	1	0.6	41.55	1.925
1 mg/l IBA in	100	100	50	3	1	1.5	0.7	41.55	1.925
2 mg/l IBA in	100	100	100	2	2	1.5	0.5	30.8	2
2 mg/l IBA in	100	100	50	2	2	0.8	0.3	30.8	2
2 mg/l IBA in	100	100	50	6	1	2	1.3	39.025	2.25
2 mg/l IBA in	100	100	50	1	1	1.3	1.6	39.025	2.25
2 mg/l IBA in	100	100	50	2	1	1.2	1.3	37.55	2.25
2 mg/l IBA in	100	100	50	3	1	1.3	0.7	37.55	2.275
4 mg/l IBA in	100	100	100	3	4	1.3	0.7	49.625	3.875

4 mg/l IBA in	100	100	0	2	2	1	0.5	49.625	3.875
4 mg/l IBA in	100	100	100	2	2	1.8	0.7	47.325	3.375
4 mg/l IBA in	100	100	50	1	1	0.8	0.9	47.325	3.375
4 mg/l IBA in	100	100	50	5	1	1.2	1	43.25	3.175
4 mg/l IBA in	100	100	50	4	1	1.6	0.8	43.25	3.175
0 mg/l in Liqu	100	100	0	0	0	0	0	0	0
0 mg/l in Liqu	100	100	0	0	0	0	0	0	0
0 mg/l in Liqu	100	100	0	0	0	0	0	0	0
0 mg/l in Liqu	100	100	0	0	0	0	0	0	0
0 mg/l in Liqu	100	100	0	0	0	0	0	0	0
0 mg/l in Liqu	100	100	0	0	0	0	0	0	0
0.5 mg/l NAA	100	100	100	4	5	1.8	0.3	51.2	5.05
0.5 mg/l NAA	100	100	100	6	1	3.7	0.3	22.7	6
0.5 mg/l NAA	100	100	100	7	4	3.3	0.9	45.35	10.3
0.5 mg/l NAA	100	100	100	3	1	2.7	0.3	46.65	8.65
0.5 mg/l NAA	100	100	50	9	2	3.6	0.2	86.9	3.35
0.5 mg/l NAA	100	100	50	7	1	4.3	1.6	51.2	5.05
1 mg/l NAA in	100	100	100	8	1	3	0.3	45.35	8.5
1 mg/l NAA in	100	100	100	4	1	2	1.3	75.95	5
1 mg/l NAA in	100	100	100	7	2	3.4	0.2	66.85	8.55
1 mg/l NAA in	100	100	100	6	1	3.2	0.8	79.35	8.6
1 mg/l NAA in	100	100	100	6	2	2.5	0.2	32.05	4.8
1 mg/l NAA in	100	100	100	4	2	2.6	0.3	66.85	8.55
2 mg/l NAA in	100	100	50	5	0	1.4	0	39.25	4.95
2 mg/l NAA in	100	100	50	5	0	2.3	0	57.85	6.25
2 mg/l NAA in	100	100	50	7	0	2.5	0	36.05	2.15
2 mg/l NAA in	100	100	50	8	0	2.1	0	19.7	5
2 mg/l NAA in	100	100	50	8	0	1.6	0	15.25	2.25
2 mg/l NAA in	100	100	50	4	0	2	0	36.05	2.15
4 mg/l NAA in	100	100	0	0	0	0	0	0	0
4 mg/l NAA in	100	100	0	0	0	0	0	0	0
4 mg/l NAA in	100	100	0	0	0	0	0	0	0
4 mg/l NAA in	100	100	0	0	0	0	0	0	0
4 mg/l NAA in	100	100	0	0	0	0	0	0	0
4 mg/l NAA in	100	100	0	0	0	0	0	0	0
0.5 mg/l IBA i	100	100	0	0	0	0	0	0	0
0.5 mg/l IBA i	100	100	0	0	0	0	0	0	0
0.5 mg/l IBA i	100	100	0	0	0	0	0	0	0
0.5 mg/l IBA i	100	100	0	0	0	0	0	0	0
0.5 mg/l IBA i	100	100	0	0	0	0	0	0	0
0.5 mg/l IBA i	100	100	0	0	0	0	0	0	0
1 mg/l IBA in	100	100	0	0	0	0	0	0	0
1 mg/l IBA in	100	100	0	0	0	0	0	0	0
1 mg/l IBA in	100	100	0	0	0	0	0	0	0
1 mg/l IBA in	100	100	0	0	0	0	0	0	0
1 mg/l IBA in	100	100	0	0	0	0	0	0	0
1 mg/l IBA in	100	100	0	0	0	0	0	0	0
2 mg/l IBA in	100	100	0	0	0	0	0	0	0
2 mg/l IBA in	100	100	0	0	0	0	0	0	0
2 mg/l IBA in	100	100	0	0	0	0	0	0	0

2 mg/l IBA in	100	100	0	0	0	0	0	0	0
2 mg/l IBA in	100	100	0	0	0	0	0	0	0
2 mg/l IBA in	100	100	0	0	0	0	0	0	0
4 mg/l IBA in	100	100	0	0	0	0	0	0	0
4 mg/l IBA in	100	100	0	0	0	0	0	0	0
4 mg/l IBA in	100	100	0	0	0	0	0	0	0
4 mg/l IBA in	100	100	0	0	0	0	0	0	0
4 mg/l IBA in	100	100	0	0	0	0	0	0	0
4 mg/l IBA in	100	100	0	0	0	0	0	0	0

Treatment	Yield	TPC	TFC	TSC	DPPH
Natural ro	16.161	1.9028	0.716679	17.9077	1752.71
Natural ro	15.606	1.9661	0.759402	18.1335	1705.81
Natural ro	15.017	1.9582	0.764742	17.9782	1766.72
0 mg/l in S	0	0	0	0	0
0 mg/l in S	0	0	0	0	0
0 mg/l in S	0	0	0	0	0
0.5 mg/l N.	12.35	0.8599	0.591478	14.9055	8759.15
0.5 mg/l N.	11.71	0.8812	0.663434	14.8294	4510.96
0.5 mg/l N.	11.88	0.8173	0.591478	14.9625	6287.5
1 mg/l in N	15.95	1.4269	0.656585	19.1857	3607.57
1 mg/l in N	15.56	1.4068	0.796572	19.0186	4602.17
1 mg/l in N	16.61	1.4269	0.810119	18.4697	5064.56
2 mg/l NA	30.47	3.9112	2.075764	33.3217	2149.17
2 mg/l NA	29.91	3.7616	2.227353	34.2297	2613.84
2 mg/l NA	31.62	3.9262	2.237459	33.9627	2106.3
4 mg/l NA	13.82	1.9583	1.092378	19.25	2594.68
4 mg/l NA	15.24	2.007	1.11588	19.4736	2920.29
4 mg/l NA	15.85	1.9583	1.064175	19.4736	2737.05
0.5 mg/l IB	30.77	6.3626	4.498389	18.2852	1580.63
0.5 mg/l IB	39.58	6.3328	4.760158	18.1788	1570.3
0.5 mg/l IB	37.25	6.2136	4.719885	17.4338	1352.48
1 mg/l IBA	28.57	4.9212	1.300642	18.0921	1764.94
1 mg/l IBA	32	4.8188	1.205516	18.0921	1771.95
1 mg/l IBA	25	4.8316	1.248755	18.3207	1685.68
2 mg/l IBA	29.67	6.3598	1.864567	18.2541	1208.51
2 mg/l IBA	34.09	6.4181	3.065608	19.1387	1213.81
2 mg/l IBA	28.09	6.1704	1.972858	19.2949	1204.23
4 mg/l IBA	30.28	5.8501	1.528883	20.2592	1297.42
4 mg/l IBA	28.99	6.0312	1.520725	21.4666	1302.03
4 mg/l IBA	27.66	5.7535	1.186231	20.0004	1299.66
0 mg/l in L	0	0	0	0	0
0 mg/l in L	0	0	0	0	0
0 mg/l in L	0	0	0	0	0
0.5 mg/l N.	43.73	8.8809	3.432313	35.7949	1016.49
0.5 mg/l N.	46.1	8.7242	2.559018	37.1937	1014.43
0.5 mg/l N.	45.36	8.6263	2.638409	36.9838	1010.44
1 mg/l NA	43.85	9.8015	2.921799	39.4744	946.074
1 mg/l NA	45.88	10.135	2.874367	39.4118	1082.91

1 mg/l NAA	47.14	9.9419	2.732072	39.1611	1106.39
2 mg/l NAA	38	9.1931	2.768625	24.0811	751.465
2 mg/l NAA	32.93	9.1931	1.987998	24.3169	755.995
2 mg/l NAA	36.6	8.907	1.824438	24.3169	752.934
4 mg/l NAA	0	0	0	0	0
4 mg/l NAA	0	0	0	0	0
4 mg/l NAA	0	0	0	0	0
0.5 mg/l IB	0	0	0	0	0
0.5 mg/l IB	0	0	0	0	0
0.5 mg/l IB	0	0	0	0	0
1 mg/l IBA	0	0	0	0	0
1 mg/l IBA	0	0	0	0	0
1 mg/l IBA	0	0	0	0	0
2 mg/l IBA	0	0	0	0	0
2 mg/l IBA	0	0	0	0	0
2 mg/l IBA	0	0	0	0	0
4 mg/l IBA	0	0	0	0	0
4 mg/l IBA	0	0	0	0	0
4 mg/l IBA	0	0	0	0	0
